# Management of ileocolic anastomotic strictures in Crohn’s disease: endoscopic or surgical intervention? A systematic review and meta-analysis

**DOI:** 10.1007/s00384-025-04958-y

**Published:** 2025-07-24

**Authors:** Mohamed Talaat Issa, Shafquat Zaman, Ali Yasen Mohamedahmed, Mohammed Hamid, Omar Mostafa, Sangara Narayanasamy, Diwakar Sarma, Rajeev Peravali, Akinfemi Akingboye, Peter Waterland

**Affiliations:** 1 Department of General and Colorectal Surgery, Dudley Group NHS Foundation Trust, Russells Hall Hospital, Dudley, West Midlands UK; 2 College of Medical and Dental Sciences, School of Medicine, University of Birmingham , Edgbaston, Birmingham, UK; 3 Department of General and Colorectal Surgery, University Hospitals of Derby and Burton NHS Foundation Trust, Queen’s Hospital Burton, Burton-on-Trent, Staffordshire UK; 4 Department of General Surgery, Wye Valley NHS Trust, Hereford County Hospital, Hereford, Herefordshire, UK; 5 Department of General and Colorectal Surgery, Sandwell and West Birmingham Hospitals NHS Trust, Sandwell General Hospital, West Bromwich,, West Midlands UK; 6 Life Sciences and College of Medicine, Aston Medical School, Aston University, Birmingham, UK

**Keywords:** Crohn’s disease, Endoscopy, Ileo-colonic disease, Inflammatory bowel disease

## Abstract

**Background:**

Intestinal strictures are one of the most intractable and common complications of Crohn’s disease (CD), and their optimal management remains debatable. Endoscopic balloon dilatation (EBD) and stricturoplasty are advanced minimally invasive therapeutic tools in the management of Crohn’s strictures and offer an alternative to surgery. We evaluated outcomes following endoscopic intervention compared with surgical resection in the management of ileocolic anastomotic strictures in patients with CD.

**Methods:**

A comprehensive and systematic search of various electronic databases was conducted. All studies comparing endoscopic intervention with surgical resection for ileocolic anastomotic strictures in patients with CD were included. Our primary outcomes were re-operation or re-dilatation post-intervention and complications including haemorrhage, perforation, leak, and surgical site infection. Other evaluated parameters included the need to escalate medical treatment following primary intervention. RevMan 5.3 was used to perform the data analysis.

**Results:**

Four observational studies with a total of 625 patients were identified and included. This consisted of 355 patients treated surgically and 270 undergoing endoscopic procedures. No significant difference in the risk of re-operation [OR, 0.13; *P* = 0.19], re-stenosis [OR, 0.58; *P* = 0.37], or total complications [OR, 1.86; *P* = 0.34] was seen between the two groups.

However, escalation of medical therapy post-intervention was significantly lower in the surgical group compared with those managed endoscopically [OR, 0.19; *P* = 0.0001].

**Conclusion:**

Both surgical and endoscopic treatments are safe and efficacious in managing patients with anastomotic strictures. However, this review emphasises the need for rationally designed, well-powered, randomised controlled trials to establish best practices in treating these challenging patients.

## Introduction

Crohn’s disease (CD) characterised by transmural inflammation in genetically susceptible individuals is associated with abnormal healing of the bowel mucosa frequently affecting the terminal ileum and leading to stricture formation [1, 2]. Despite advances in medical management including steroid therapy, biologics, and tumour necrosis factor (anti-TNF) inhibitors, the management of CD-related intestinal strictures remains a challenge, and often these agents are used as a temporary measure to improve or reduce bowel inflammation and therefore symptoms [3].

Historically, a significant proportion (approximately 71%) of these patients required surgical intervention and bowel resection in the first 10 years following diagnosis. Unfortunately, there is a relatively high rate of recurrence of symptoms over time, estimated to be 36% for symptomatic patients [4]. As a consequence of disease burden, ileocecal resection with ileocolic anastomosis is a common surgical procedure performed in medically refractory cases [5].

However, a sequela of such intervention is the occurrence of anastomotic strictures (AS), occurring in about 40% of patients with CD post ileocolic anastomosis [6, 7]. These patients often present with symptoms of complete or partial bowel obstruction, with a significant adverse impact on quality of life.

Managing AS is challenging and optimal treatment remains debatable. Unlike inflammatory strictures, AS usually require mechanical intervention and modalities include endoscopic therapies or various surgical platforms (open, laparoscopic, robotic) [5]. Endoscopic interventions are comparatively less invasive and can be used as a ‘bridge’ to defer/delay surgery. Moreover, surgical intervention is associated with long-term adhesions and hernia occurrence, in addition to immediate and short-term risks (bleeding, leak, collections/abscess formation) [6, 8].

Endoscopic interventions can be performed through various approaches: balloon dilatation (EBD), endoscopic stricturotomy (ESt), endoscopic stricturoplasty, steroid injections, and self-expanding metal stents (SEMS). Each of these is associated with its own risks and varying success rates and necessitates careful patient selection. A randomised controlled trial (RCT) reported EBD delaying surgical intervention for 6.5 years [9]. ESt showed immediate complete technical success in patients with AS and a subsequent surgical resection rate of 15.3% [10].

Moreover, endoscopy is associated with relatively fewer complications compared with major surgical resections but is not considered a permanent or lasting treatment. It often needs multiple repeat sessions and is confounded by the risk of perforation and bleeding each time [11]. Surgery for AS tends to offer a longer period of remission (symptom-free).

The aim of the present study was to compare outcomes between endoscopic and surgical management of ileocolic AS in patients with CD. To our knowledge, this is the first meta-analysis of the available published literature on this topic.

## Methods

### Study design and PROSPERO registration

The Preferred Reporting Items for Systematic Review and Meta-analysis (PRISMA) guidelines [12, 13] were followed in designing, performing, and reporting this review and meta-analysis. The study was registered in the International Prospective Register of Systematic Reviews (PROSPERO) reference no: CRD42024546073 (available at http://www.crd.york.ac.uk/prospero).

### Sources of data and search strategy

 Two authors performed the literature search independently. The search included multiple electronic databases and clinical trial registries: Cochrane, Embase, PubMed, Science Direct, Scopus, Clinical Trials.gov, and Virtual Health Library (VHL). The last search was performed on 20/05/2024 using the following terms: “Crohn’s disease”, “Crohns disease”, “terminal ileitis”, “ileocolic resection”, “ileocecal resection”, “right hemicolectomy” OR “bowel resection” AND “terminal ileum stricture”, “anastomotic stricture”, “post-anastomotic stricture”, “terminal ileum stenosis”, “anastomotic stenosis”, “post-anastomotic stenosis” AND “endoscopic procedure”, “endoscopic intervention”, “ballon dilatation”, “endoscopic balloon dilatation”, “stenting”, “stent”, “stricturoplasty”, “endoscopic stricturoplasty” AND “stenosis resection”, “bowel resection for stenosis”, “bowel resection for stricture”. Additionally, a manual search of references was conducted to identify further relevant studies.

### Article selection

The identified studies’ titles, abstracts, and full texts were screened independently by two authors to find potentially eligible studies. All studies comparing any surgical resection for AS with any endoscopic therapy were judged. Case reports, single-arm studies, case series, letters to the editor, conference abstracts, and review articles were excluded. Any disagreements arising at this stage between the reviewers on inclusion and exclusion criteria were resolved through discussion with the authorship team.

### Data extraction and collection

Two authors independently extracted data from selected articles and recorded this by populating onto a Microsoft Excel sheet. The recorded data were as follows: year of publication, name of first author, country of study, total number of patients, patient demographics, and study outcomes. Any disagreement between the data extraction team was dealt with by consulting the wider authorship team and reaching an agreement.

### Outcome measures

Our primary outcomes post-initial intervention were rates of re-intervention and re-stenosis and post-intervention complications. The evaluated secondary outcome measure was the need to escalate medical treatment for CD following index intervention between the two groups.

### Bias assessment

In this meta-analysis, all the included studies were observational by design and no RCTs were included. Consequently, the Cochrane Risk of Bias Tool [14] for assessing RCTs was not utilised. The risk of bias was assessed through a star-based scoring system as per the Newcastle–Ottawa Scale (NOS) [15]. Scoring of 9, 7/8, or less than 6 was graded as low, medium, or high risk of bias, respectively. Any disagreement was rectified through discussion with a third author.

### Statistical analysis

Odds ratio (OR) was used as the statistical measure for dichotomous outcomes using the Mantel–Haenszel method. The *P* value and 95% confidence interval (CI) were calculated for all analyses, and a *P* value was considered statistically significant if it was less than 0.05. Random-effects modelling was applied to all outcome analyses.

Moreover, ‘between-study heterogeneity’ was assessed using the I^2^ and χ^2^ statistic. The study was considered to have a high level of heterogeneity with high reported values of I^2^. I^2^ values exceeding 50% were considered significant heterogeneity [16].

To check for possible causes of heterogeneity and evaluate the robustness of our results, sensitivity analysis was performed by calculating the risk ratio (RR) or risk difference (RD) for dichotomous variables. Moreover, a leave-one-out analysis was conducted to assess each study’s effect individually.

Review Manager 5.0 (Nordic Cochrane Centre, Cochrane Collaboration) was used for all statistical analyses.

## Results

The initial search of the aforementioned electronic databases identified 245 studies. Following a review of titles and abstracts, 134 studies were excluded from the subsequent analysis. Full manuscripts of the remaining 111 studies were reviewed and evaluated against the eligibility criteria, identifying four relevant studies [9, 17–19] included in our final analysis.

**Fig. 1:**
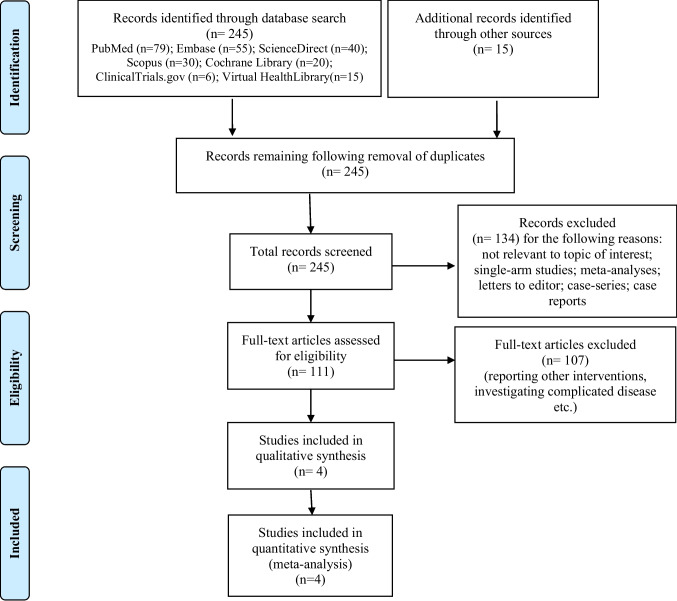
PRISMA flow chart

PRISMA flow chart is illustrated in Fig. 1.

All identified studies were retrospective cohort by design with a total of 625 patients. These were divided as follows: 355 patients underwent surgery and 270 had an endoscopic intervention.

Table 1 shows characteristics of the included studies, and Table 2 illustrates patients’ demographics. The median length of stricture was 7 cm in the surgical group (SG) and 3 cm in the endoscopic group (EG).

**Table 1:** Characteristics of the included studies

Study	Country	Type of Study	Population	Inclusion and exclusion criteria	Follow-up
Lian [9]	USA	Retrospective cohort	SG: 131 EG: 176	**Inclusion criteria:** CD patients having either 1) EBD for symptomatic ICA stricture or 2) upfront bowel resection for ICA stricture **Exclusion criteria:** patients < 18 years of age or having dilations for the primary, non-anastomotic CD strictures; patients with concurrent enterocutaneous fistula, abdominal abscess or other definitive indications for surgical intervention; patients with asymptomatic ICA strictures and with surgical stricturoplasty for ICA strictures; patients with non-CD ICA strictures; no patients were included in both EBD and surgery groups	**Median:** **SG**: 4.7 years (IQR, 2.2–8.8) **EG**: 1.8 years (IQR, 0.4–4.1)
Krauss [17]	Germany	Retrospective cohort	SG: 37 EG: 20	**Inclusion criteria:** patients undergoing either surgical or endoscopic therapy for symptomatic intestinal strictures **Exclusion criteria:** inflammatory processes in the immediate proximity of the intestine to be dilated or resected (intestinal perforation, fistulas, abscesses, peritonitis)	**Mean (range):** 5 years (4–8)
Greener [18]	Israel	Retrospective cohort	SG: 40 EG: 39	**Inclusion criteria:** **SG:** CD with obstructive symptoms that led to the stricture, stricturing CD with or without complications (abscess or sinus), and resection of the affected segment **EG:** CD with obstructive symptoms that were attributed to the stricture by the treating physician, a documented non traversable stricture on endoscopy, and endoscopic dilation **Exclusion criteria:** **SG:** intestinal resections with an indication other than intestinal stricture **ED:** patients with dilations for non-CD indications	**Mean (SD:** **SG:** 3.5 ± 2.5 years **EG:** 2.8 ± 1.7 years
Lan [19]	USA	Retrospective cohort	SG: 147 EG: 35	**Inclusion criteria:** primary diagnosis of CD with 1 or more prior ileocolonic bowel resections, stricture at ICA site, and ICA strictures treated with either ESt or ICR **Exclusion criteria:** patients with a diagnosis of non-CD conditions; non-ICA stricture, which is a primary (i.e. disease-associated) stricture located in other parts of the bowel; concurrent penetrating disease at the time of stricture diagnosis; or inception procedure performed in outside hospitals	**Median:** **SG:** 2.2 years (1.2–4.4) **EG**: 0.8 years (0.2–1.7)

*SG* surgical group, *EG* endoscopic group, *CD* Crohn’s disease, *EBD* endoscopic balloon dilatation, *ICA* ileocolonic anastomosis, *ESt* endoscopic stricturotomy, *IQR* inter-quartile range

**Table 2:** Patient demographics

	Lian [9]		Krauss [17]		Greener [18]		Lan [19]	
	SG *n* = 131	EG *n* = 176	SG *n* = 37	EG *n* = 20	SG *n* = 40	EG *n* = 39	SG *n* = 147	EG *n* = 35
Age (years) Mean (range/SD)	45.4 ± 11.6	43 ± 13.2	44.6 (22–83)	39.4 (19–63)	27.9 ± 12.8	23.2 ± 12.3	48.6 ± 13.4	42.6 ± 15.5
Male (%)	60 (45.8)	94 (43.4)	17 (45.9)	9 (45)	24 (60)	27 (69)	80 (54.4)	24 (68.6)
Caucasian (%)	127 (96.9)	161 (91.5)	NA	NA	NA	NA	133 (97.1)	34 (90.5)
Mean CD disease duration (years) (range/SD)	19 ± 9.5	17.3 ± 10.5	11 (1–20)	12.5 (11–18)	12 ± 6.3	16.5 ± 11.7	NA	NA
Operation-free time	9.9 ± 7.5	9.8 ± 8.4	Median: 69 months (55–83)	Median: 52 months (39–71)	NA	NA	NA	NA
Symptom-free time	NA	NA	Median: 68 months (55–83)	Median: 21 months (5–44)	NA	NA	NA	NA
Previous surgery (%)	66 (50.4)	64 (36.4)	50 (ileocecal resection: 74; right hemicolectomy: 16)	55 (ileocecal resection: 64; right hemicolectomy: 18; subtotal colectomy: 9)	2 (5)	20 (52)	NA	NA
Current smoker (%)	77 (58.8)	33 (18.8)	NA	7 (35)	NA	NA	35 (23.8)	2 (5.7)
Former smoker (%)	5 (3.8)	44 (25)	NA	NA	NA	NA	50 (34)	10 (28.6)
Never smoked (%)	49 (37.4)	99 (56.2)	NA	NA	NA	NA	62 (42.2)	23 (65.7)
Immunosuppressants/Steroids prior to intervention (%)	44 (33.6)	33 (18.8)	16 (44.7)	6 (30)	8/30 (27)	3/30 (10)	58 (39.5)	12 (34.3)
Biologics prior to intervention (%)	29 (22.1)	31 (17.6)	NA	1 (3)	9/30 (30)	19/36 (52)	44 (29.9%)	18 (51.4)

*SD* standard deviation, *CD* Crohn’s disease, *SG* surgical group, *EG* endoscopic group, *NA* not available

### Risk of bias assessment

Table 3 outlines the outcomes of the methodological quality assessment as per the NOS for observational studies.

**Table 3:** Risk of bias assessment as per the Newcastle–Ottawa Scale

Study	Representativeness of the exposed cohort	Selection of the non-exposed cohort	Ascertainment of exposure	Demonstration that outcome of interest was not present at start of the study	Comparability of cohorts based on the design or analysis controlled for confounders	Assessment of outcome	Was follow-up long enough for outcomes to occur	Adequacy of follow-up of cohorts	Total
Lian [9]	*	*	*	*	*	*	*	*	8
Krauss [17]	*	*	*	*		*	*	*	7
Greener [18]	*	*	*	*		*		*	6
Lan [19]	*	*	*	*	*	*	*	*	8

## Outcomes

### Primary

#### Re-intervention

**Fig. 2:**
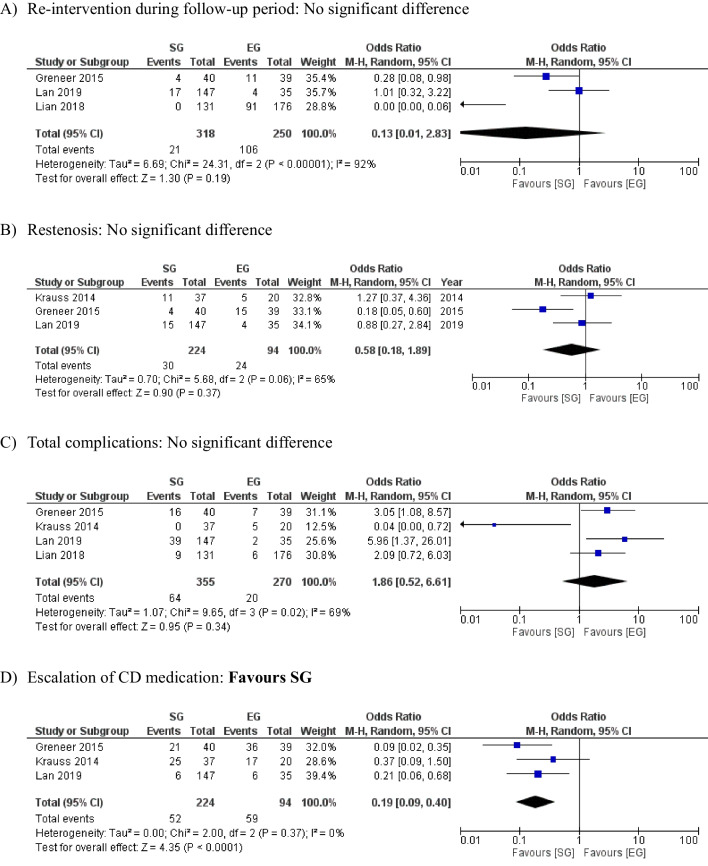
Forest plots of comparison of (**A**) reintervention during follow-up period, **B** restenosis, **C** total complications, and (**D**) escalation of CD medication. The solid squares denote the odds ratio. The horizontal lines represent the 95% confidence intervals (CIs), and the diamond denotes the pooled effect size. SG, surgical group; EG, endoscopic group; CD, Crohn’s disease; M–H, Mantel–Haenszel test

The measured outcome was the need for re-operation or re-endoscopy following the index procedure. This was higher in the SG but failed to reach statistical significance (6.6% SG vs. 4.2% EG), OR 0.13, 95% CI (0.01, 2.83), *P* = 0.19. A substantial risk of between-study heterogeneity was observed using the Cochran Q test (I^2^ = 92%; *P* < 0.00001) (Fig. 2A).

#### Re-stenosis

Re-stenosis at the site of anastomosis was evaluated between the two groups. This was 13.4% in the SG and 25.5% in the EG, respectively, with no statistically significant difference, OR 0.58, 95% CI (0.18, 1.89), *P* = 0.37. A moderate risk of between-study heterogeneity was observed using the Cochran Q test (I^2^ = 65%; *P* = 0.06) (Fig. 2B).

#### Major complications

Major post-procedure complications following surgery and endoscopy are shown in Tables 4 and 5, respectively. As these were not directly comparable, the rates of total complications occurring following each intervention have been used instead. These were 18% in the SG and 7.4% in the EG, OR 1.86, 95% CI (0.52, 6.61), *P* = 0.34. A moderate risk of between-study heterogeneity was observed using the Cochran Q test (I^2^ = 69%; *P* = 0.02) (Fig. 2C).

**Table 4:** Postoperative complications in the surgical group

Postoperative complication	Lian [9] (*n* = 131) (%)	Krauss [17] (*n* = 37) (%)	Greener [18] (n = 40) (%)	**Lan** [19] **(*n* = 147)** **was rectified through discussion**	**Total** **(*n* = 355)** **(%)**
Anastomotic leak	0 (0)	0 (0)	2 (5)	2 (1.4)	**4 (1.2)**
Pelvic abscess	5 (3.8)	0 (0)	4 (10)	9 (6.1)	**18 (5.1)**
Enterocutaneous fistula	4 (3.1)	0 (0)	1 (2.5)	0 (0)	**5 (1.4)**
Surgical Site infection	0 (0)	0 (0)	8 (20)	11 (7.5)	**19 (5.4)**
Ileus	0 (0)	0 (0)	0 (0)	17 (11.6)	**17 (4.8)**
Iatrogenic injury	0 (0)	0 (0)	1 (2.5)	0 (0)	**1 (0.3)**

**Table 5:** Post-procedure complications in the endoscopic group

Post-procedure complication	Lian [9] (*n* = 176) (%)	Krauss **[**17] (*n* = 20) (%)	Greener [18] (*n* = 39) (%)	Lan [19] (*n* = 35) (%)	Total (*n* = 270) (%)
Perforation	2 (1.1)	5 (25)	0 (0)	1 (2.9)	**8 (3%)**
Bleeding	0 (0)	0 (0)	2 (5.1)	5 (14.3)	**7 (2.6)**
Fever with abdominal pain (no perforation)	0 (0)	0 (0)	3 (7.7)	0 (0)	**3 (1.1)**
Prolonged hospitalisation	0 (0)	0 (0)	2 (5.1)	0 (0)	**2 (0.7)**
Technical failure	17 (9.7)	0 (0)	7 (18)	1 (2.9)	**25 (9.3)**

### Secondary

#### Escalation of post-intervention medications for CD

Escalation of CD medications was reported in three studies with a total of 318 cases. The SG showed a significantly lower medication escalation rate compared  with the EG (23.2% vs. 62.8%), OR 0.19, 95% CI (0.09, 0.40), *P* = 0.0001. There was a low level of heterogeneity between the included studies. (I^2^ = 0%, *P* = 0.37) (Fig. 2-D). 

### Sensitivity analysis

The direction of the pooled effect size remained unchanged when the RR or RD was calculated for dichotomous variables. Furthermore, the leave-one-out analysis did not reveal any significant discrepancies with the original analysis.

## Discussion

We compared outcomes following surgical resection and endoscopic interventions in the management of AS in patients with CD. We believe this is the first review and meta-analysis synthesising best available data from mainly low-quality studies.

No notable differences were seen between the two comparison arms for post-intervention major complications (such as bleeding or perforation). The two approaches also showed no significant difference in re-stenosis or re-operation rates. However, escalation of medical treatment post index procedure for CD was significantly lower following conventional surgical treatment (*P* = 0.0001).

Approximately 80% of patients undergoing ileocaecal resection with ileocolic anastomosis for CD require at least one further surgical resection in the following decade [20]. AS is a frequent complication and often needs mechanical intervention, either surgical or endoscopic. Although the exact aetiology of stricture formation postoperatively is poorly understood, a combination of Crohn’s recurrence and surgical factors increases the risk of strictures at the site of anastomosis [21, 22].

Endoscopic therapy, although relatively novel, is effective in selected patients. Lian et al. compared EBD with surgery in treating AS for CD patients (who had previously undergone ileocaecal resection). Endoscopic intervention showed a lower adverse event rate compared with surgical resection but was associated with a higher need for subsequent surgery compared with the surgically treated cohort [9]. A 2–3% risk of perforation was reported in a review of 1163 patients undergoing EBD for CD strictures [23].

Although EBD delayed the need for surgery by approximately 6.5 years, the need for subsequent surgery was lower in patients undergoing surgical resection [9]. Stenosis recurrence rates post-endoscopic intervention in patients following ileo-colic resection and anastomosis are reportedly high (up to 70% in the first 1–1.5 years post-intervention) [24].

ESt can be used to relieve strictures along the GI tract [25–27], as well as treat stricture formation in ileal pouches [28], ileorectal anastomosis [29], continent ileostomy [30], and colostomies [31]. Lan et al. reported a decreased need for surgery post-ESt and a perforation rate of 4% [10]. The need for future surgery and perforation rate was lower in ESt compared to EBD (9% vs. 33%) and (0% vs. 1%), respectively [10]. However, despite the superiority of ESt as a treatment option over EBD, ESt is technically demanding with higher rates of bleeding [10].

Surgical resection of AS provides symptomatic benefit but may lead to repeated surgery [5, 32, 33]. Ileo-colic resection for stenosis was associated with a 10% (5 years) and 17.5% (10 years) risk of re-intervention at follow-up [34]. The present study showed no statistically significant difference between surgical and endoscopic intervention with regard to re-operation/re-endoscopy and re-stenosis.

Moreover, surgical resection requires a prolonged hospital stay with considerable financial implications, balanced against the need for multiple endoscopic interventions [11]. Endoscopic intervention is advocated by some as it reduces the need for multiple bowel resections and the potential development of short bowel syndrome. However, patients with extensive small bowel disease may not be suitable candidates for endoscopic intervention [18].

Traditionally, surgery has also been associated with an increased risk of major complications compared with endoscopic procedures. However, this was not evident in our review and meta-analysis.

Previous studies [17–19] comparing surgical and endoscopic intervention to treat AS in CD patients reported that surgical resection was associated with a lower use of CD-related medications and avoided its escalation for more extended periods postoperatively than post-endoscopic therapy. We also show similar findings and found that the need for escalation of medication was lower in those patients undergoing resection.

However, choice of intervention is multi-factorial and includes taking into consideration amongst other things, the length of intestinal stricture, duration of symptoms, and nature of stenosis (inflammatory vs. fibrotic) [35].

Ouali et al. [35] found stricture length to predict the need for surgery in follow-up at 12, 24, and 48 months (*P* = 0.03). Bamba et al. [36] conducted a multi-centre study and reported stricture length < 2 cm was associated with less surgical intervention. A previous systematic review by Hassan et al. [37] reported strictures < 4 cm were associated with a surgery-free outcome. Bettenworth et al. [38] found an increase in stricture length by 1 cm increased the risk of surgery by 8%.

The role of radiographic assessment of these strictures and disease severity is well established within the literature [39, 40]. Felley et al. [41] found EBD to be effective in treating fibro-stenotic strictures less than < 5 mm. Krauss et al. [17] reported no difference in observed operation-free and symptom-free times between inflamed and fibrotic strictures [*P* = 0.805, *P* = 0.237] in an endoscopically treated cohort. Therefore, endoscopic intervention has a critical role in establishing stricture type and concomitant role with medical management in providing symptomatic relief in inflammatory narrowing.

Stenosis can often lead to and act as a cause of bowel obstruction. Medical management may not be appropriate at this late stage and surgery is often warranted to provide symptomatic relief. Surgery and endoscopic procedures are two separate strategies for treating AS. However, select patients may benefit from a combination of approaches. For instance, high-risk, malnourished patients may get temporary benefits from an initial minimally invasive approach. Endoscopic intervention can act as a useful ‘bridging’ mechanism in managing CD patients presenting with AS. It delays the time to surgery, allowing patient optimisation and elective rather than emergent surgery. A technical success rate of 86% for EBD was reported, with long-term efficacy of 58% (over 33-month follow-up period) [23]. Therefore, endoscopy procedures potentially offer a window for patient optimisation and may reduce the risk of anastomotic leak and/or the need for stoma formation.

This study is not without its limitations which need to be addressed. High heterogeneity was present in some of our analyses, and the sensitivity analysis was unable to reduce this. Potential causes for the observed high heterogeneity may be attributed to a multitude of factors including (but not limited to) differences in study populations/demographics (ethnicity, age at diagnosis, co-morbidities, disease characteristics, environmental exposure); study design; variation in outcome measures/criteria and data collection; and even geographical and regional differences*.* Secondly, the quality-of-life indicators and metrics post-intervention were not assessed. These are equally important measurements of symptom control and success of intervention performed. Thirdly, we were unable to measure the short and long-term cost-effectiveness between the different intervention groups from the available data. Cost-effectiveness analysis is an integral and important consideration in healthcare systems in utilising finite and often limited resources to achieve best possible health-related outcomes. A recent microsimulation state-transition model analysed risks and benefits of surgery and EBD for patients with primary or anastomotic CD strictures [42]. They concluded that EBD was a cost-effective strategy and suitable alternative for managing CD strictures. However, careful patient selection ideally through an MDT setting taking into account local availability and expertise with endoscopic treatment modalities is important. Moreover, future studies are needed to determine the cost-effectiveness and long-term efficacy of different treatment strategies. Fourthly, no RCT studies and therefore data were available on the topic of interest, reducing the quality of data synthesis. Furthermore, this meta-analysis did not account for variations in length of intestinal strictures, presence of inflammation within the strictures, or frequency of balloon dilatations performed; all which would influence management choice. Finally, due to a lack of available data, we were unable to compare individual endoscopic interventions (EBD or ESt) as a sub-group with surgical resection.

## Conclusion

Current evidence from low-quality studies suggests that endoscopic intervention and conventional surgical resection in managing ileocolic anastomotic strictures in patients with CD have comparable outcomes; both are safe and efficacious. Surgical resection seems to be superior in reducing the need for escalating medical therapy post-intervention. Endoscopic techniques in carefully selected cases could act as a useful ‘bridge’ in delaying the need for more invasive treatment. This review emphasises the need for rationally designed, well-powered, randomised controlled trials to compare these approaches and establish best practice.

## Data Availability

No datasets were generated or analysed during the current study.
